# Protocol for Systematic Review: “Understanding Climate Sensitive Infectious Disease Burden in Australia”

**DOI:** 10.12688/f1000research.178008.1

**Published:** 2026-04-06

**Authors:** Gabriel Parker, Fan Yu, Claire F Brereton, Nina Lansbury, Nicolas Smoll, Benn Sartorius

**Affiliations:** 1 School of Public Health, Faculty of Health, Medicine and Behavioural Sciences, The University of Queensland, Herston, QLD, Australia; 2 The University of Queensland School of Public Health, Herston, Queensland, Australia; 3 Sunshine Coast Public Health Unit, Queensland Health, Maroochydore, QLD, Australia

**Keywords:** Infectious diseases; Communicable diseases; Climate change; Global warming; Australia

## Abstract

Climate change is increasingly being recognized as a major driver of infectious disease risk globally, and timely information is required to support public health policy decision-makers. This protocol aims to provide local policy makers and scientists with a framework for conducting rapid and timely systematic reviews to estimate how short/long-term climate changes influences the burden and geographical distribution of human infectious diseases in Australia. Using the Joanna Briggs Institute (JBI) Population, Exposure, Outcome (PEO) methodology, the systematic review outlined in this protocol will include observational and modelling studies from 1995–2025 that examine climate– infectious disease relationships in human populations in all regions (urban/rural and remote) of Australia. Searches are to be conducted across several databases (PubMed, Web of Science, Scopus, Embase, CINAHL, Informit, Google Scholar). Data extraction shall use concise eligibility criteria to capture study design, exposure metrics, outcome measures, statistical models, and effect measures. The review shall standardize exposures on different scales (e.g., per 1 °C or per 10 mm rainfall) for comparability and apply log transformations for meta-analysis where feasible. Risk of bias for studies included in the review will be assessed using JBI critical appraisal tools relevant to the study type being evaluated. Synthesis, with meta-analysis and meta-regression is to be considered if sufficient comparable data exists. If synthesis of data is feasible the ROBINS-E tool for risk of bias shall be applied for studies included in the meta-analysis/regression. This protocol provides the framework for the conduct of a systematic literature review that will inform public health policy that relates to climate-sensitive infectious diseases in Australia by synthesizing evidence on associations between both short-term variability of weather/climatic factors (temperature, rainfall, humidity, and extremes) as well as longer term climate change and infectious diseases in humans in Australia.

PROSPERO ID: CRD420251268644.

## Introduction

### Rationale

Despite ongoing and urgent global calls for decisive climate action, 2024 saw increased greenhouse gas emissions and an exceedance of 1.5 °C in the mean annual temperature above preindustrial levels for the first time. ^1^ Current projections provide a compelling case that the global climate will continue to undergo significant warming in response to ongoing emissions of greenhouse gases (GHG) to the atmosphere. ^2^ Anthropogenic Climate Change including changes in temperature, rainfall, and extreme weather are associated with increased frequency and spread of diseases in wildlife, agriculture, and humans. ^3^ The link between climate change and human infectious disease (ID) is widely recognised. More than half (58%) of human pathogenic diseases can be aggravated by climate change ^4^ and environmental condition changes are affecting vector-borne, waterborne, foodborne, airborne, and soilborne disease transmission. ^1^ In recent times there has been an intensifying call for countries to place health at the forefront of their national responses to climate change. ^5^

Australia is a large continent country with a very diverse range of climatic zones (from alpine to tropical). ^6^ It faces increased disease risks in face of ongoing climate change due to several changes including rates of spread and multiplication of pathogens, numbers, range and activity of vector species (e.g. mosquitoes, ticks, sandflies) that transmit some infectious diseases and numbers, range and activity of non-human host species (e.g. rodents, bats) that naturally harbour zoonotic agents able to cause disease in humans. ^7^

In recent times Australia has faced outbreaks of diseases identified as being climate sensitive. For example, in 2021/2022 the emergence of Japanese Encephalitis Virus (JEV) in more temperate Southeast Australia created concern in both community and public health circles as it had not previously been a major disease issue on the continent, outside of a few sporadic cases in the far tropical north. Climate variability, including La Niña-associated heavy rainfall, has been recognised as a contributing factor to this spread and increase in number of JEV cases. ^8^ Climate change factors have also been identified as a driver for increased cases of vector-borne diseases such as Ross River virus, ^9^ foodborne diseases such as salmonellosis, ^10^ gastrointestinal diseases such as cryptosporidiosis, ^11^ soil-borne diseases such as melioidosis ^12^ and zoonotic diseases such as leptospirosis. ^13^

To date there has not been a systematic review examining the evidence describing and quantifying the overall effect of short-term variability of weather/climatic factors (temperature, rainfall, humidity, and extremes) as well as longer term climate change across all climate sensitive infectious disease pathways in people (humans) living in Australia as well as attempting to quantify effect sizes and non-linear effects. This protocol allows the researcher to create a systematic review that will provide timely insights into the climate factors that drive infectious disease risks in Australia.

## Aim

### Research objectives

Systematically identify and quantify evidence on:

• Associations between short-term meteorological variability and human infectious disease burden in Australia. (NB Short-term meteorological variability refers to weather and climate fluctuations occurring from days to interannual timescales (<30 years), including heatwaves, rainfall anomalies, rapid humidity changes, and drought). ^14^
• Associations between longer-term climate change indicators or trends and human infectious disease burden in Australia. (NB: Long-term climate indicators refer to multi-decadal trends (≥30 years) in climatic conditions, such as long-term warming, precipitation shifts, and climate-model–derived projections.) ^14^

To quantify the relationships between short- and long-term climatic factors (and change therein) and infectious disease burden in Australia, with the goal of:

• Determining which IDs are most sensitive to climate variability and change
• Characterising the nature and strength of these associations across Australia's diverse climatic regions (tropical, arid, temperate, and alpine), population types (urban, rural, and remote), and priority subgroups including Indigenous (First Nations) Australians.
• Characterising the nature and strength of these associations on the incidence and/or related burden (e.g. hospitalisation or mortality).
• Evaluating the methodological quality and certainty of the evidence to inform public health surveillance, modelling, and climate adaptation strategies.

### Research questions

What are the associations between climatic factors (specifically short-term meteorological variability and long-term anthropogenic climate change) and a) incidence of climate-sensitive infectious diseases in humans in Australia, and b) occurrence, magnitude, and duration of infectious disease outbreaks and associated burden (hospitalisation, mortality) in Australia?

Which diseases show the strongest and most consistent associations with specific climatic exposures (e.g., temperature, rainfall, humidity, extreme weather events)?

What is the overall certainty of evidence regarding climate sensitivity for different infectious diseases in the Australian context?

How do these climate ID associations vary by geographic region (e.g., tropical vs temperate zones), population subgroups (e.g., First Nations, rural/remote communities), and disease transmission pathways (e.g., vector-borne, waterborne, foodborne)?

What methodological frameworks or approaches have been developed and applied to assess climate–disease relationships in Australia, and what are the strengths and limitations of these approaches?

## Protocol methods

The methods used in this systematic review protocol adhere to the Preferred reporting items for systematic review and meta-analysis protocols (PRISMA-P) 2015. ^15^ Completed PRISMA-P 2015 checklist available via the authors Git Hub repository. ^16^

This protocol is registered with the International Prospective Register of Systematic Reviews (PROSPERO ID: CRD420251268644) **.** Any amendments following registration will be documented in the PROSPERO record, including the date, description of the change, and rationale.

## Eligibility criteria

### Study designs

Observational analytic studies (time-series, case-crossover, cohort, case–control, ecological analyses), and modelling studies using surveillance and meteorological/environmental data; exclude pure opinion/commentary and reviews (systematic/literature/narrative).

### Inclusion & Exclusion criteria

See Table 1 for detailed inclusion and exclusion criteria.

**Table 1.** Inclusion/exclusion criteria for review.

Inclusion	Exclusion
1. Population - Australia (and Australian external territories).	Population - Not relevant to Australia (or Australian external territories).
2. Exposure - Short Term Meteorological Variables (e.g. Precipitation, Temperature, humidity) or Longer-Term Anthropogenic Climate Change Factors (e.g. increased temperatures over decades).	Exposure - Not related to short-term Meteorological Variables (e.g. Precipitation, Temperature, humidity) or Longer-Term Anthropogenic Climate Change Factors (e.g. increased temperatures over decades).
3. Outcome - Infectious or Contagious Diseases.	Outcome - Not discussing Infectious or Contagious Diseases.
4. Studies on (or impact on) Humans.	Non-Human Studies.
5. Written in English.	Non-English.
6. Full text.	Not full text, only abstract.
7. Published in peer reviewed journal as full research article.	Not published in peer reviewed journal as full research article.
8. Articles focused on climate effects on ID transmission in Australia. Can analyse one or many diseases or methods of analysis or modelling/forecasting: Core review and Frameworks. Role of Climate Change in ID emergence, re- emergence, or worsening. Vector-Borne Disease Studies (Ross River Virus, Dengue, etc.) Zoonotic, Food, Soil & Water-Borne Diseases Infectious diseases with multiple or other transmission pathways e.g. person to person.	Not relevant to Australian context (NB: International papers with broad climate sensitive infectious disease contexts will be considered for inclusion if Australian data clearly stratified). Article focused on ID’s without examining climate change effects. Systematic/Literature/Narrative Reviews.
9. Date range: 1st January 1995 to 31st October 2025.	Published outside the specified date range.
10. Operationalised approaches, frameworks, models, tools, guidance material.	Records consisting solely of background/overview material without operationalised methods.

## Information sources

PubMed, Web of Science Scopus, Embase, CINAHL, Informit, & Google Scholar & AI Prompts.

## Search criteria

### Example search strategy

Database: PubMed.

Limits: Dates 1995–2025, Full Text, English only.

(“Communicable Diseases”[Mesh] OR “Disease Transmission, Infectious”[Mesh] OR infectious disease*[tiab] OR communicable disease*[tiab] OR transmissible disease*[tiab] OR contagious disease*[tiab] OR infection*[tiab] OR pathogen*[tiab] OR zoonos*[tiab] OR vector-borne [tiab] OR respiratory disease*[tiab] OR foodborne disease*[tiab] OR water-borne disease*[tiab] OR food-borne disease*[tiab] OR soil transmitted disease*[tiab] OR person to person transmission [tiab])

AND

(“Climate Change”[Mesh] OR “Global Warming”[Mesh] OR climate change*[tiab] OR global warming [tiab] OR climate crisis [tiab] OR climate emergency*[tiab] OR climate variability [tiab] OR environmental change*[tiab] OR extreme weather [tiab] OR temperature*[tiab] OR temperature variability [tiab] OR precipitation OR rainfall OR “humidity”[MeSH Terms] OR humidity [tiab] OR “droughts”[MeSH Terms] OR drought*[tiab] OR global heating [tiab] OR heat exposure [tiab] OR heatwave*[tiab] OR climate projection*[tiab])

AND

(Australia [tiab] OR “Australia”[Mesh] OR Australia*[tiab] OR Australas*[tiab] OR “New South Wales”[tiab] OR “Victoria”[tiab] OR “Queensland”[tiab] OR “South Australia”[tiab] OR “Western Australia”[tiab] OR “Tasmania”[tiab] OR “Northern Territory”[tiab] OR “Northern Australia”[tiab] OR “Australian Capital Territory”[tiab] OR “tropical Australia” [tiab] OR “remote Australia”[tiab])

**NB:** Full data base search terms available via the authors Git Hub repository. ^16^

## Study selection process

The systematic review shall use the Covidence tool for article screening and study selection. ^17^

### Initial screening

Articles extracted by the search should de-duplicated prior to screening. Once de-duplication has been completed two independent investigators shall review a small sample (200) of extracted articles before the full selection process is completed. To ensure consistency piloting terms should be recorded by both reviewers. All remaining titles and abstracts shall be independently assessed against the inclusion criteria and categorized as either ‘included’ or ‘excluded’. Disagreements shall be resolved through discussion, and any unresolved conflicts are to be adjudicated by a third investigator. After completion of the title and abstract screening stage, inter-rater reliability is to be assessed using Cohen’s kappa (κ). ^18^ A κ value of ≥0.60 shall be considered acceptable (substantial agreement). If κ falls below this threshold, eligibility criteria will need to be refined, and additional reviewer calibration is to be undertaken before proceeding to full-text screening.

### Full-text review

A full-text review of those studies that are deemed to fit the inclusion criteria is to be undertaken by two independent investigators to determine if they are indeed appropriate for inclusion in the review. Again, in cases of disagreements between the two reviewers the articles shall be evaluated and resolved by a third independent investigator. Once this process is complete all included studies move into the data extraction phase.

### Data item extraction

The following information shall be extracted and recorded in duplicate spreadsheets by two independent reviewers. In the case of data being unavailable in the selected studies, the authors of the studies will be contacted to request the data.

### Data items

**Study information –** title, authors, date of publication, journal, DOI and abstract.

**Study design –** for example time-series, case-crossover, cohort or ecological designs.

**Study setting –** urban/rural; tropical/temperate.

**Time period –** for example years.

**Seasonality handling –** for example season fixed effects.

**Population –** Australia (Country/State/External Territory/City).

**Exposure type –** Climate change differentiated into:

1. Short-term meteorological variability and extreme events: precipitation, temperature, humidity, floods/heavy rainfall, droughts, fires, heatwaves, vegetation cover e.g. Normalized Difference Vegetation Index (NDVI) and sudden changes to vegetation cover due to storms or fires.
2. Long term secular changes: Mean annual temperature trends, long-term precipitation patterns, long term humidity trends and long-term vegetation cover changes (e.g. Normalized Difference Vegetation Index changes (NDVI) over time).

**Exposure specification –** metric, lag structure, thresholds, extreme definitions, data source BoM, reanalysis of climate variables.

**Outcome type –** Infectious diseases sensitive to short term meteorological variations or long-term climate change affecting humans in Australia. Multiple disease classifications (e.g. air, vector, soil, water and food borne) will be considered.

**Outcome source & case definition –** National Notifiable Diseases Surveillance System (NNDSS), hospital admissions, lab-confirmed definitions.

**Adjustments –** confounders: population density, Socio Economic Status (SES), age, First Nations status where reported, mobility, interventions, vector control.

**Statistical methods used** -

1. Statistical models – including commonly used regression-based and time-series frameworks such as Generalised Additive Models (GAMs), Poisson or negative binomial regression, Distributed Lag Non-Linear Models (DLNMs), and Autoregressive Integrated Moving Average (ARIMA) models.
2. Mechanistic or deterministic models – encompassing compartmental models (e.g., SIR/SEIR models), vector-population dynamic models, or climate-driven transmission models that explicitly represent underlying biological or ecological processes governing pathogen or vector dynamics.
3. Machine-learning and ensemble modelling approaches – including algorithms such as random forests, gradient boosting machines, support vector machines, artificial neural networks, and hybrid ensemble methods that use data-driven techniques to model potentially complex, nonlinear relationships between climate exposures and disease incidence.

**Effect measure –** e.g. Incidence Rate Ratio (IRR) increase per °C or per 1 mm rainfall increase.

**Standardization –** e.g. convert effect sizes to common units or scale (e.g. normalised z-scores) for different outcome measure (see data synthesis below).

### Data management

All records and data related to this systematic review shall be managed using a combination of EndNote for reference management and Covidence for screening, data extraction, and quality assessment. Search results from databases (e.g., PubMed, Embase) are to be exported in RIS format and imported into EndNote for deduplication. The deduplicated records shall then be uploaded to Covidence, where two independent reviewers are to screen titles, abstracts, and full texts. Data extraction will be conducted in duplicate using a piloted form within Covidence. All data is to be stored securely on a password-protected cloud storage (e.g. university or government agency), with access restricted to the research team.

## Methodology

This is primarily an etiological question. Therefore, studies will be selected under the JBI Population/Exposure of Interest/Outcome or Response (PEO) criteria. ^19^

1) Population = People in Australia (including external territories).

2) Exposure of interest = Climatic factors (a) short-term meteorological variability and extremes (heatwaves, rainfall anomalies, humidity, drought) and/or (b). long-term climate change indicators (e.g., trend in temperature/precipitation, climate projections),

3) Outcome or response = Burden of infectious diseases (incidence, prevalence, hospitalization, mortality, outbreak occurrence), by disease category (vector-, water-, food-, soil-borne, respiratory/person-to-person).

## Data synthesis and analysis

### Risk of bias assessment

If synthesis of data from subgroups of papers e.g. those discussing climate variables effects on vector or water borne diseases is possible then the JBI Critical Appraisal Tool appropriate for the study being examined (e.g. Checklist for Prevalence Studies) ^20^ or ROBINS-E ^21^ will be utilised to assess for risk of bias. Results of these tools will be reported in the review plus used in any stratified sensitivity analyses/synthesis.

### Data synthesis

Meta-analysis will only be conducted when a minimum of five studies contribute comparable effect estimates to a given pooled analysis, with five or more preferred for increased stability. Heterogeneity will be quantified using the I ^2^ statistic, and where substantial heterogeneity is observed, subgroup analyses or meta-regression will be explored as prespecified.

**Narrative synthesis –** If quantitative pooling is inappropriate due to considerable heterogeneity (defined as I ^2^ ≥ 75%, following Deeks et al.) ^22^ or incompatible effect measures (e.g. where exposure response shapes or parameterisations differ), findings will be synthesised narratively by comparing patterns across disease types, climate variables, study designs, and geographic contexts.

**Quantitative analysis –** The JBI guide describes statistical synthesis of quantitative results from two or more studies as meta-analysis. ^23^ However this protocol specifies a pragmatic threshold of >5 studies for the feasibility of conducting a meta-analysis thus stabilising statistical analysis and allowing heterogeneity metrics to be interpretable. In addition, exposure standardisation (convert to same contrasts e.g. per 1 °C or normalised to z-score) will be conducted as will lag handling, and DLNM extraction (e.g. relative risk percentile comparison (50 vs 90), cumulative vs specific optimal lags). If summaries are able to be reduced and pooled using multivariate meta- analysis DLNM studies will be quantitively analysed. Non linear exposure-response relationships (e.g. threshold effects, U or J shaped associations and spline-based associations) will be handled according to their original form. Study results are to be categorised into either short-term meteorological variability and extremes (heatwaves, rainfall anomalies, humidity, drought) or annual secular long-term climate change trends (e.g. temperature/precipitation, climate projections).

Effect estimates (e.g., risk ratios, odds ratios, incidence rate ratios) shall be pooled using a random-effects meta-analysis framework. To improve the accuracy of uncertainty estimates, particularly when the number of contributing studies is small, Hartung–Knapp adjustments will be applied to derive confidence intervals. In addition to pooled estimates, 95% prediction intervals are to be calculated to reflect the expected range of true effects across different settings. Where studies contribute multiple dependent effect sizes (e.g., several climate variables, multiple lag structures, or multiple outcomes derived from the same dataset), robust variance estimation (RVE) shall be used to account for within-study correlation and prevent overweighting of studies providing several related effects.

**Additional analyses –** If sufficient data are available, additional analyses is to be undertaken to enhance the robustness and interpretability of findings. Where appropriate sensitivity analysis assessing the robustness of findings, including leave-one-out analyses, exclusion of high-risk-of-bias studies, evaluation of alternative climate-exposure definitions, and exclusion of studies contributing disproportionately to heterogeneity (I ^2^ ≥ 75%) shall be conducted. Subgroup analyses will explore potential sources of heterogeneity by stratifying results according to Bureau of Meteorology (BoM) climate zones (e.g., tropical, subtropical, temperate, arid), disease category (such as vector-borne versus water-borne infections), geographic region, and specific climate variables (temperature, rainfall, humidity). Where appropriate, meta-regression is to be conducted to examine whether study-level characteristics (including design, time period, population demographics, or climate exposure metrics) account for variability in effect sizes.

Where there is feasibility for meta-analysis, meta-bias shall be assessed through visual inspection of funnel plots and statistical tests such as Egger’s test and Kendall’s τ. Selective reporting within individual studies shall be evaluated during the risk of bias assessment using validated tools such as JBI or ROBINS-E. The certainty of evidence across studies for each outcome is to be appraised using the GRADE (Grading of Recommendations, Assessment, Development and Evaluation) framework.

**Dissemination –** Findings from the review are to be disseminated through publication in a peer-reviewed journal and presentation at relevant scientific conferences and seminars. Given the applied public health relevance of the review, results are to be communicated to appropriate health and government stakeholders to support evidence-informed decision-making and potential implementation. This may include public health units, environmental health agencies, or other organisations responsible for climate and infectious disease preparedness. Dissemination pathways have been considered during the design of the review to maximise usability and uptake, while ensuring the protocol remains broadly applicable for future investigators conducting similar reviews.

## Amendment tracking

All amendments to this protocol will be recorded in an Amendments Log (Excel spread sheet) and presented as an appendix in the Systematic Review final report. The log will contain the following elements:

○ **Version Number** (e.g., V1.1) in which document amendment occurred
○ **Date of Amendment**
○ **
Section Modified** (e.g., “Search Strategy,” “Eligibility Criteria”)
○ **Nature of Change** (e.g., “Added a new database to search,” “Narrowed the scope to exclude single-family studies”)
○ **Justification** (e.g., “Preliminary search revealed a high volume of irrelevant studies”).

## Ethical considerations

There are no ethical considerations as this is a review of publicly available peer reviewed research articles.

## Guarantor statement

Gabriel Parker is the guarantor for this paper and accepts full responsibility for the overall content. He has access to all the data and controlled the decision to publish.

## Study status

Ongoing - Current phase: Title & Abstract screening.

## Data Availability

Repository: Git Hub (doi via Zenodo). Git Hub checklist title: “ extended_data_for_sr_protocol_understanding_csids_burden_in_australia ”. Link: https://github.com/G-Parker-82/extended_data_for_sr_protocol_understanding_csids_burden_in_australia/tree/main/extended-data doi: https://doi.org/10.5281/zenodo.18691241 ^16^ Licence: CC0 1.0 Universal (CC0 1.0). The review will follow the Preferred Reporting Items for Systematic Reviews and Meta-Analyses (PRISMA) guidelines. The results will be presented in tables summarizing key findings, risk of bias assessment, and the geographic distribution of the included studies. doi: https://doi.org/10.5281/zenodo.18691241 ^16^ Licence: CC0 1.0 Universal (CC0 1.0).
