# Supplementary material for: Protocol for Systematic Review: “Understanding Climate Sensitive Infectious Disease Burden in Australia”
Source: F1000Res. 2026 Apr 6;15:475. [Version 1] doi: 10.12688/f1000research.178008.1 (PMC13216778; doi:10.12688/f1000research.178008.1)
Supplement: Peer review history

# Reviewer report: Reviewer response for version 1

**DOI:** 10.5256/f1000research.196339.r480731

**Published:** 2026-05-27

**Authors:** Damià Barceló

**Affiliations:** 1 University of Almeria, Almería, Spain

The protocol for a systematic review on climate sensitive infectious diseases looks good to me. The authors will conduct a systematic literature review in the Australian context. All the steps are clearly presented including study design, time period and population among other parameters.

 Just one comment to add-

- I suggest to revise critically all meta-data analysis from the literature and select the most appropriate papers. In this respect I would like to add that different conclusions can be obtained from population studies of few hundred individuals compared to pilot studies of 30-5o individuals. Please make clear in your paper which ones are comprehensive exposure studies and which ones are pilot studies.

Is the study design appropriate for the research question?

Yes

Is the rationale for, and objectives of, the study clearly described?

Yes

Are sufficient details of the methods provided to allow replication by others?

Yes

Are the datasets clearly presented in a useable and accessible format?

Yes

Reviewer Expertise:

environmental chemistry and toxicology

I confirm that I have read this submission and believe that I have an appropriate level of expertise to confirm that it is of an acceptable scientific standard.

# Reviewer report: Reviewer response for version 1

**DOI:** 10.5256/f1000research.196339.r483679

**Published:** 2026-05-13

**Authors:** Ioannis Pantelis Adamopoulos

**Affiliations:** 1 Hellenic Republic Region of Attica, Athens, Greece

The protocol is well established in all sections and also very importantly registered in PROSPERO; it only concerns about 5 studies included in the meta-analysis. I suggest re-evaluating the inclusion criteria and importing more studies, up to 10-12. In this type of study, there are thousands of studies that must be checked in all databases (PubMed, Scopus, WoS, etc.).

Is the study design appropriate for the research question?

Yes

Is the rationale for, and objectives of, the study clearly described?

Yes

Are sufficient details of the methods provided to allow replication by others?

Yes

Are the datasets clearly presented in a useable and accessible format?

Yes

Reviewer Expertise:

PUBLIC HEALTH, CLIMATE CRISIS MANAGEMENT, ENVIRONMENTAL HEALTH, OSH

I confirm that I have read this submission and believe that I have an appropriate level of expertise to confirm that it is of an acceptable scientific standard.
